# Isocitrate Dehydrogenase *IDH1* and *IDH2* Mutations in Human Cancer: Prognostic Implications for Gliomas

**DOI:** 10.3389/bjbs.2021.10208

**Published:** 2022-01-31

**Authors:** A. K. Murugan, A. S. Alzahrani

**Affiliations:** ^1^ Department of Molecular Oncology, King Faisal Specialist Hospital and Research Centre, Riyadh, Saudi Arabia; ^2^ Department of Medicine, King Faisal Specialist Hospital and Research Centre, Riyadh, Saudi Arabia

**Keywords:** *IDH1*, *IDH2*, mutation, cancer, glioma, dehydrogenase, isocitrate

## Abstract

**Background:** There are isolated reports of mutations in genes for isocitrate dehydrogenases (*IDH1* and *IDH2*), but few have been examined in a large number of different malignancies. We aimed to analyze mutational prevalence of these genes in a large series of cancers and determine their significance in most mutated phenotype.

**Methods:** We analyzed the frequencies of *IDH1* and *IDH2* mutations in 14,726 malignancies of 37 cancers. Furthermore, we examined these mutations in the most frequent cancer (gliomas, 923 cases) from a single cohort, and determined their clinical significance.

**Results:** *IDH1* mutations were present in 3% (473/14,726) of cancers. The highest frequencies were in oligodendrogliomas (91/102, 89%), anaplastic oligodendrogliomas (40/46, 87%), and diffuse astrocytomas (89/116, 77%). *IDH2* mutation was detected in <1% (83/14,726) of cancers, but were present in 13% (6/46) of anaplastic oligodendrogliomas, 9% (9/102) of oligodendrogliomas, and in 5% (2/39) of cutaneous squamous cell carcinomas. Further analyses of 923 gliomas revealed 34 and 1% of *IDH1* and *IDH2* mutations, respectively. In up to 342 months of follow-up, *IDH1* and *IDH2* mutations were significantly linked with better overall (OS) (both *p* = 0.01) and progression-free survival (PFS) (*p* = 0.01; *p* = 0.004), respectively.

**Conclusion:** *IDH1* and *IDH2* are often mutated in a tissue-specific manner, most commonly in gliomas. Mutation in both genes is linked to OS and PFS. Our findings suggest that these genes are promising therapeutic targets and strong prognostic biomarkers in gliomas.

## Introduction

Malignancy is the most common non-communicable disease and accountable for one in eight deaths across the globe. The incidence of cancer and cancer-related death for the year 2021 has been projected to occur in 1,898,160 and 608,570 cases, respectively in the United States. The cancer-related death rate has significantly fallen since 2017, accounting for an overall decline of 29% that is equal to 2.9 million fewer deaths. Over the past decade, the death rate was shown to be significantly decreased in the leading cancers including lung, colorectal, breast, and prostate nevertheless, the decline slowed in breast and colorectal cancers of females, and the decline stopped in prostate cancer (1). Various next-generation transcriptomic, genomic, and proteomic studies identified many genetically deregulated genes in human cancer. These altered cancer gene clusters exert deregulated signaling on certain pathways as somatic mutations in receptor tyrosine kinases (RTKs) and their downstream pathway members including different RAS (H/K/N) molecules constitutively trigger canonical mitogen-activated protein kinase (MAPK) and phosphatidylinositol 3-kinase (PI3K) signaling. Particularly, high frequent genetic alterations of various genes like *TP53*, *BRAF*, *RAS*, *EGFR*, *PIK3CA*, *PTEN*, *HER2*, *UDX*, *ALK*, *TERT*, *mTOR*; *IDH1*, etc. were documented in the oncogenesis of several kinds of human malignancies (2–17). Aberrant activation of the vital pathways promotes uncontrolled cell division, proliferation, growth, invasion, and metastasis that collectively leads to tumorigenesis.

Isocitrate dehydrogenase 1 (*IDH1*) gene mutations were high frequently detected in human cancers particularly in secondary glioblastomas (>70%) (18). IDH1 mutations are present mainly in the hotspot arginine at codon R132 and many different *IDH1* mutations (R132H, R132S, R132C, and R132G) were also reported for the residue R132. The *IDH2* mutations were identified in codon 172 and malignancies with no mutations in *IDH1* frequently showed mutations in the cognate amino acid arginine (R) at 172 of the *IDH2* gene. All the codon R132 *IDH1* mutants were shown to decrease the enzymatic activity of the IDH1 (16). Frequent mutations of the *IDH1* have also been identified in hormone receptor-positive (HR+) breast adenocarcinoma, thyroid cancer, cholangiocarcinoma (50–70%), and *IDH2* (R172S) in benign giant cell tumors of the bone (80%) (17, 19–22).

IDH plays a key role within the Krebs cycle and produces alpha-ketoglutarate (α-KG) by catalyzing the oxidative decarboxylation of isocitrate. The IDH activity is exclusively dependent on nicotinamide adenine dinucleotide phosphate (NADP+) which is catalyzed by IDH1 to produce NADPH that is involved in controlling oxidative damage of a cell (23). The cancer-associated *IDH1* mutations have been demonstrated to produce 2-hydroxyglutarate as this IDH (IDH1 and IDH2) mutant enzyme carries a neomorphic catalytic function and converts alpha-ketoglutarate to 2-hydroxyglutarate that suppresses the histone lysine demethylases (23–25). It has been reported that an *IDH1* mutation was potentially able to form glioma hypermethylation phenotype while *IDH2* could promote acute myeloid leukemia (26). Nevertheless, a prominent status of the *IDH1* and *IDH2* mutations has never been undertaken particularly within a large number of different human cancer samples.

Given the importance of these genes, we investigated the *IDH1* and *IDH2* mutations in a large series of cancer cases (*n* = 14,726) (solid malignancies) from the data of The Cancer Genome Atlas and the Memorial Slone Kettering Cancer Centre.

## Methods

As detailed in Table 1, we analyzed 15,300 malignant tumour samples (obtained from 14,726 patients) of 37 different types of malignancies in solid tissue cancers. All data was derived from The Cancer Genome Atlas studies by performing various analyses using the methods within the cBioPortal (www.cbioportal.org), an open-access, open-source, and publicly available platform for interactive exploration of multidimensional cancer genomics datasets. Approval statement/informed consent is not required for this study as we used data from a publicly available database.

**TABLE 1:** Human cancer samples (solid tumors) analyzed for *IDH1* and *IDH2* gene mutations.

S. No	Name of organ	Type of cancer	Number of samples
1	Adrenal gland	Adrenocortical carcinoma	92
2	Ampulla of vater	Ampullary carcinoma	160
3	Biliary tract	Colangiocarcinoma	195
4	Bladder	Bladder cancer	413
5	Bowel	Colorectal adenocarcinoma	619
6	Breast	Breast cancer	2,509
7	Brain	Glioma	1,004
8		Glioblastoma	543
9		Medulloblastoma	125
10	Cervix	Cervical squamous cell carcinoma	297
11	Esophagus	Esophageal squamous cell carcinoma	139
12		Esophageal carcinoma	559
13		Gastric adenocarcinoma	78
14		Metastastic esophagogastric cancer	341
15		Esophageal adenocarcinoma	182
16	Stomach	Stomach adenocarcinoma	440
17	Eye	Uveal melanoma	80
18	Head and Neck	Head and neck squamous cell carcinoma	523
19		Oral squamous cell carcinoma	40
20		Nasopharygeal carcinoma	56
21	Kidney	Kidney renal clear cell carcinoma	446
22	Liver	Hepatocellular Adenoma	46
23		Hepatocellular carcinomas	243
24	Lung	Small cell lung cancer	120
25		Non-small cell lung cancer	447
26	Ovary	Ovarian serous cystadenocarcinoma	489
27	Pancreas	Pancreatic adenocarcinoma	456
28	Peripheral nervous system	Pediatric neuroblastoma	1,089
29	Pleura	Pleural mesothelioma	22
30	Prostate	Metastatic prostate adenocarcinoma	444
31		Prostate adenocarcinoma	1,465
32	Skin	Basel cell carcinoma	293
33		Cutaneous squamous cell carcinoma	39
34		Metastatic melanoma	110
35		Skin cutaneous melanoma	448
36	Testis	Germ cell tumors	180
37	Thymus	Thymoma	123
38	Uterus	Uterine corpus endometrial carcinoma	373
39		Uterine carcinosarcoma	57
40	Vulva	Squamous cell carcinoma of the vulva	15
		Total	15,300^a^

a Derived from 14,726 patients.

We explored the cBioPortal database for mutational analysis of *IDH1* and *IDH2*. In brief, firstly we selected one/two cohorts of larger sample size against each malignancy from the different cohorts (cancer studies) available in the datasets (accessible from the homepage of cBioPortal). In total, 40 different cohorts were selected (users have the option to select one or more than one cohort). Secondly, we selected only “mutation” under the genomic profile menu (on the same page). Other genomic profiles including the copy number variants (CNVs), structural variant, RNAseq, etc., were excluded (unselected). Thirdly, we selected “samples with mutation data” under the patient/case set menu. Fourthly, we input *IDH1* or *IDH2* under the “enter genes menu” (users have options to enter multiple genes) and queried the *IDH1*/*IDH2* against the selected set of malignancies. The query generated a newer window with multiple tool tab options such as oncoprint, cancer types summary, plots, mutations, survival, etc., to visualize the results against various parameters of selected cohorts. Users may perform different analyses of selected datasets by selecting and submitting the intended (any of the above-indicated) tool tab. We utilized the tool tab “cancer types summary” to visualize and obtain the prevalence of *IDH1*/*IDH2* mutations in various selected malignancies. Complete and comprehensive step-by-step procedures are clearly described previously for mining the cBioPortal database (27).

The Memorial Sloan Kettering Cancer Centre data of 1,004 glioma samples (derived from 923 patients) was analyzed for *IDH1* and *IDH2* mutations within the cBioPortal (www.cbioportal.org), and clinical links including overall survival (OS) and progression-free survival (PFS) was performed by selecting the tool tab “survival tab,” excluded the overlapping samples and results were visualized as described earlier (27).

Statistical genomic analyses were executed utilizing the methods incorporated within the cBioPortal (www.cbioportal.org) (27). Kaplan–Meier plots with a log-rank test were performed to determine the OS and PFS of gliomas presence of a minimum of one mutation or absence of mutation in the related queried candidate gene. *p* < 0.05 was regarded as statistically significant.

## Results

The composition of the 15,300 different cancer samples and their malignant types are shown in Table 1. As detailed in Table 2, overall, 3% (473/14,726) of solid tumour cancer cases harbored mutations in *IDH1*. The highest frequencies were present in oligodendrogliomas, anaplastic oligodendrogliomas, and diffuse astrocytomas. The overall prevalence of *IDH2* mutation was <1% (83/14,726). The highest frequencies were present in anaplastic oligodendrogliomas, oligodendrogliomas, and cutaneous squamous cell carcinomas. These results clearly indicate that *IDH1* and *IDH2* are often mutated in solid cancers in a tissue-specific manner and the mutational incidence was frequent mainly in gliomas, suggesting that *IDH1* and *IDH2* may play important roles in these types of solid human cancer.

**TABLE 2:** Mutational prevalence of *IDH1* and *IDH2* genes in various human cancers.

Type of cancer	Mutational prevalence of *IDH1* *^a^*	Type of cancer	Mutational prevalence of *IDH2* *^a^*
Oligodendroglioma	89.2% (91/102)	Anaplastic Oligodendroglioma	13.0% (6/46)
Anaplastic Oligodendroglioma	87.0% (40/46)	Oligodendroglioma	8.8% (9/102)
Diffuse Astrocytoma	76.7% (89/116)	Cutaneous Squamous Cell Carcinoma	5.1% (2/39)
Anaplastic Astrocytoma	54.1% (86/159)	Skin Cancer, Non-Melanoma	3.4% (10/293)
Intrahepatic Cholangiocarcinoma	29.1% (46/158)	Lung Squamous Cell Carcinoma	2.6% (4/155)
Glioblastoma Multiforme	5.6% (45/796)	Intrahepatic Cholangiocarcinoma	2.5% (4/158)
Extrahepatic Cholangiocarcinoma	5.4% (2/37)	Uterine Endometrioid Carcinoma	2.0% (4/200)
Cutaneous Squamous Cell Carcinoma	5.1% (2/39)	Colorectal Adenocarcinoma	1.8% (11/619)
Cutaneous Melanoma	4.9% (27/550)	Uterine Carcinosarcoma	1.7% (1/57)
Skin Cancer, Non-Melanoma	3.7% (11/293)	Diffuse Astrocytoma	1.7% (2/116)
Esophagogastric Adenocarcinoma	2.5% (2/80)	Tubular Stomach Adenocarcinoma	1.3% (1/79)
Uterine Serous Carcinoma	2.3% (1/44)	Anaplastic Astrocytoma	1.3% (2/159)
Bladder Urothelial Carcinoma	2.2% (9/412)	Esophagogastric Adenocarcinoma	1.2% (1/80)
Colorectal Adenocarcinoma	2.1% (13/619)	Hepatocellular Carcinoma	1.2% (3/243)
Adenocarcinoma of the Gastroesophageal Junction	1.7% (1/57)	Esophagogastric Cancer	1.1% (4/347)
Uterine Endometrioid Carcinoma	1.5% (3/200)	Esophageal Squamous Cell Carcinoma	0.9% (3/324)
Stomach Adenocarcinoma	1.3% (4/308)	Esophageal Adenocarcinoma	0.76% (2/303)
Tubular Stomach Adenocarcinoma	1.3% (1/79)	Glioblastoma Multiforme	0.5% (4/796)
Esophagogastric Cancer	1.1% (4/347)	Bladder Urothelial Carcinoma	0.5% (2/412)
Thymoma	0.8% (1/123)	Cervical Squamous Cell Carcinoma	0.4% (1/247)
Head and Neck Squamous Cell Carcinoma	0.8% (4/515)	Prostate Adenocarcinoma	0.4% (7/1909)
Ampullary Carcinoma	0.6% (1/160)	Cutaneous Melanoma	0.4% (2/550)
Esophageal Squamous Cell Carcinoma	0.6% (2/324)	Stomach Adenocarcinoma	0.3% (1/308)
Renal Clear Cell Carcinoma	0.5% (2/426)	Head and Neck Squamous Cell Carcinoma	0.2% (1/515)
Prostate Adenocarcinoma	0.4% (8/1909)		

a % (Mutated samples/Analyzed total samples) of the indicated cancer type.

**FIGURE 1:**
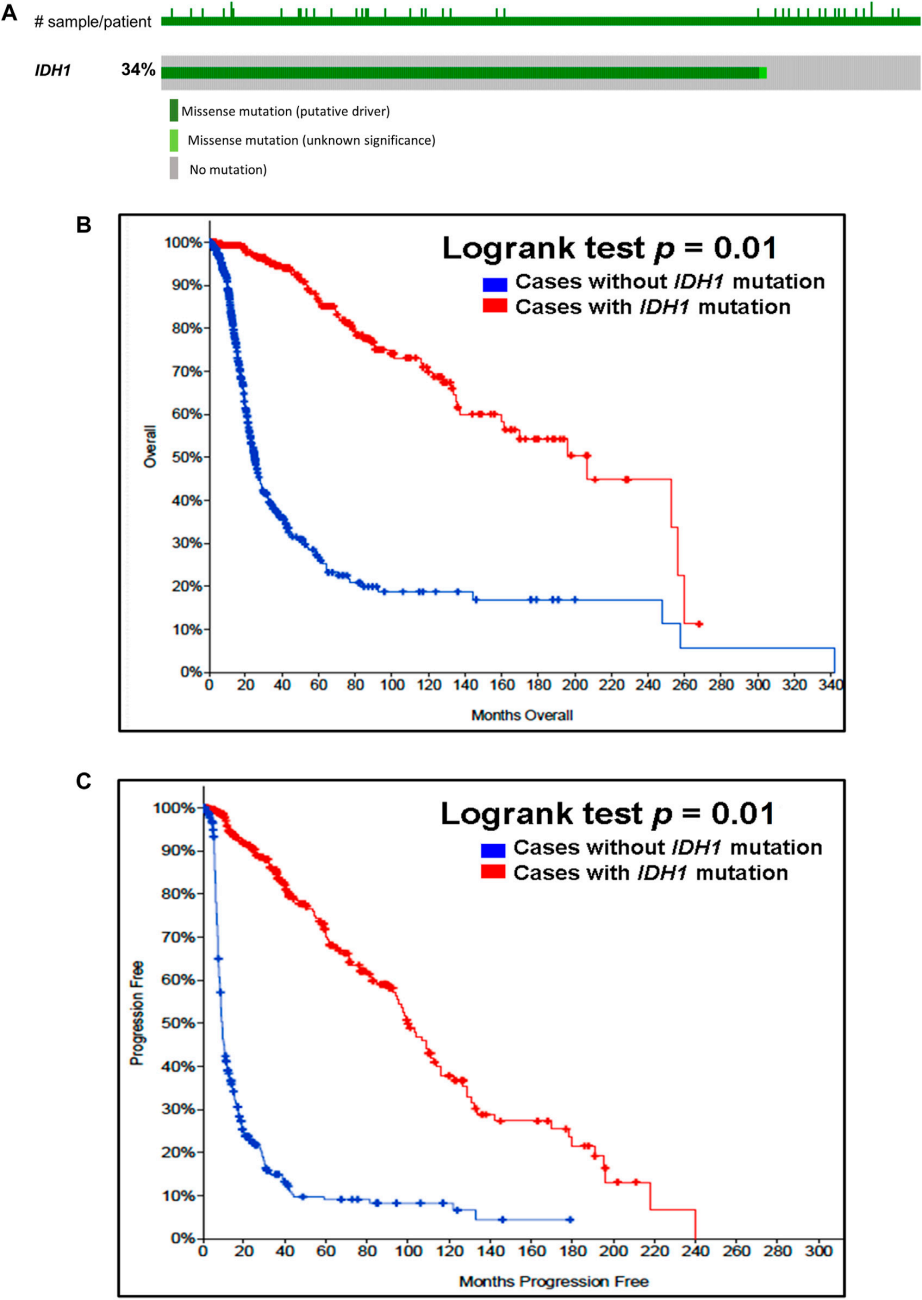
Prevalence and prognostic significance of *IDH1* mutations in gliomas **(A)** *OncoPrint tab*. The tab shows the *IDH1* mutations identified in gliomas. The row indicates the *IDH1* gene and each column show a tumor sample. The green squares plotted on the columns show non-synonymous somatic mutations. **(B)** *Overall survival curve*. Of 923 glioma cases, 2 patients were excluded from survival analysis due to overlap. The total number of patients included in the overall survival analysis = 921. Number of cases with *IDH1* mutation = 312 (number of events = 64; median overall survival (months) = 207). Number of cases without *IDH1* mutation = 609 (number of events = 280; median overall survival (months) = 25), *p* = 0.01. **(C)** *Progression-free survival curve*. The total number of patients included in the overall progression-free survival analysis = 622. Number of cases with *IDH1* mutation = 302 (number of events = 116; median progression-free survival (months) = 100). Number of cases without *IDH1* mutation = 320 (number of events = 262; median progression-free survival (months) = 9), *p* = 0.01. Diagrams **(B,C)** display the Kaplan–Meier plot of overall survival and progression-free survival of glioma patients in the absence or presence of the *IDH1* mutations which is indicated in blue and red colour, respectively.

**FIGURE 2:**
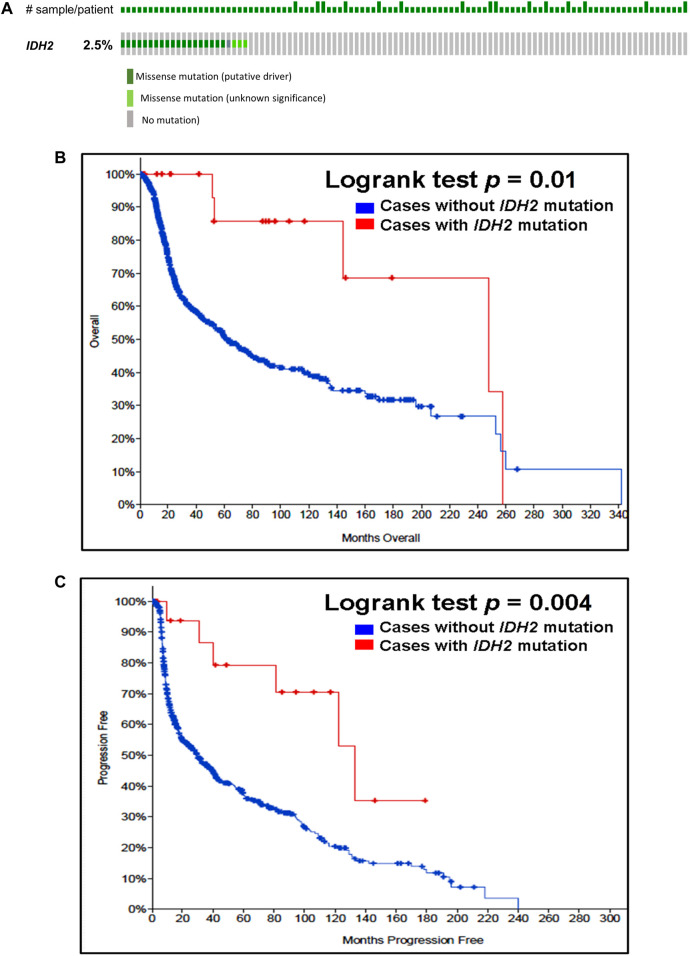
Prevalence and prognostic significance of *IDH2* mutations in gliomas **(A)** *OncoPrint tab*. The tab shows the *IDH2* mutations found in gliomas. The row indicates the *IDH2* gene and each column shows a tumor sample. The green squares plotted on the columns show non-synonymous somatic mutations. **(B)** *Overall survival curve*. The total number of patients included in the overall survival analysis = 923. Number of cases with *IDH2* mutation = 23 (number of events = 5; median overall survival (months) = 248). Number of cases without *IDH2* mutation = 900 (number of events = 339; median overall survival (months) = 62), *p* = 0.01. **(C)** *Progression-free survival curve*. The total number of patients included in the overall progression-free survival analysis = 623. Number of cases with *IDH2* mutation = 20 (number of events = 6; median progression-free survival (months) = 133). Number of cases without *IDH1* mutation = 603 (number of events = 372; median progression-free survival (months) = 30), *p* = 0.004. Diagrams **(B,C)** display the Kaplan–Meier plot of overall survival and progression-free survival of glioma patients in the absence or presence of the *IDH2* mutations which are indicated in blue and red colour, respectively.

Our analysis of cancer data from The Cancer Genome Atlas revealed a high incidence of *IDH1* and *IDH2* mutations in subtypes of gliomas. Thus, to determine the importance of these gene mutations in glioma, we analyzed them in a single and a large cohort of glioma from the Memorial Sloan Kettering Cancer Centre data sets and also examined the association of mutations of these genes in the prognosis of gliomas. As seen in Figure 1A, *IDH1* harbored somatic mutations overall in 34% (314/923) of gliomas, whilst its presence significantly predicts an improved overall survival (OS) and better progression-free survival (PFS) (Figures 1B,C). The *IDH2* mutation was detected overall in 2.5% (23/923) of gliomas (Figure 2A). The *IDH2* mutation-bearing patients were also statistically significantly associated with improved OS and better PFS (Figures 2B,C).

## Discussion

*IDH1* and *IDH2* are recurrently mutated in several types of human cancer (15–17, 19–22). Particularly, point mutations of these genes are major therapeutic targets and important prognostic markers in brain tumors (15, 16). Nonetheless, the prevalence of these mutations has not been analyzed in a large number of different types of human cancer despite the availability of next-generation sequencing data. Therefore, we mined the human cancer data derived from 37 different types of cancers, finding a high rate of *IDH1* and *IDH2* mutations in a tissue-specific manner mainly in gliomas suggesting a role for these genes in carcinogenesis. Our analysis revealed that the overall prevalence of *IDH1* mutations in cancer was 3%. The oligodendrogliomas showed a high rate of *IDH1* mutation, followed by anaplastic oligodendrogliomas and diffuse astrocytomas (all > 75%). In contrast, the overall prevalence of *IDH2* mutation was 1%, being most frequent in anaplastic oligodendrogliomas, oligodendrogliomas and cutaneous squamous cell carcinomas (<15%). Even though a number of studies were combined and cases were different in each study in our analysis, *IDH1* or *IDH2* mutational frequencies of our study reflect previously published results (16).

Having found a high frequency of *IDH1* and *IDH2* mutations in subtypes of gliomas, we hypothesised role in long-term follow-up studies, finding that both *IDH1* and *IDH2* mutations are strong prognostic factors, supporting other data (15, 16). Importantly, it has been shown that *IDH* mutations are an independent prognostic marker of favorable outcomes (28). Thus, *IDH* mutations have been shown to be associated with longer survival, unlike a mutated *TP53* cases (29). The *IDH1* mutation has been shown to be a stand-alone favorable prognostic element in low-grade oligodendrogliomas (LOs), anaplastic oligodendrogliomas (AOs) particularly when the *TP53* is not overexpressed (30). Moreover, a study investigated the prognostic value of *IDH* mutations in 99 secondary high-grade gliomas revealed that an *IDH* mutation did not associate with increased PFS although secondary anaplastic glioma patients with *IDH* mutation showed a significantly improved outcome (31). A previous study performed a meta-analysis of *IDH1* and *IDH2* mutations from 55 different studies with 9,487 glioma tumors found both mutations were independently and statistically significantly associated with better OS and PFS of glioma patients (32). Analyses of 24 different studies displayed that glioma patients with *IDH* mutations were associated with improved OS and PFS (33). These results provide additional evidence that collectively indicates the important roles of these genes and suggests that *IDH* mutations are strong prognostic markers for survival in gliomas. Conversely, recently it has been shown that the median overall survival from the first progression was not significantly different between the IDH1 mutant and wild-type group when primary and secondary glioblastomas were combined. On the other hand, the median overall survival from the initial diagnosis was significantly different (34). These findings clearly indicate that *IDH1* and *IDH2* could serve as potential independent diagnostic and prognostic biomarkers in these malignancies.

*IDH1* and *IDH2* are promising molecular targets for precision therapy not only in gliomas but also in other malignancies. Consistent with this notion, the mutant IDH1 (ivosidenib) and IDH2 (enasidenib) protein inhibitors have been initially approved by the U.S. food and drug administration (FDA) for relapsed/refractory acute myeloid leukemia (AML)-bearing *IDH1* and *IDH2* mutations (35). Subsequently, IDH1 inhibitor was also approved for newly diagnosed cases of AML, and currently the drug is being clinically evaluated for other cancers including cholangiocarcinoma with *IDH1* mutation (35, 36). Furthermore, the *IDH1*-mutated tumors were recently targeted by a vaccine that exhibited vaccine-mediated tumor response in the majority of cases (37). Particularly, the FDA has already approved the mutant *IDH1* and *IDH2* test and hence, these findings can be expanded by testing *IDH1* and *IDH2* mutations in different malignancies in both the diagnostic phase and during the course of treatment to examine if the mutation evolves so that the tumors harboring *IDH1* and *IDH2* mutation could benefit from the *IDH1/2*-mediated targeted therapy. These advances collectively demonstrate that the *IDH1* and *IDH2* mutations play a key role in the therapeutic determination of gliomas and a subset of other malignancies.

In conclusion, we identified a high incidence of *IDH1* and *IDH2* mutations in a tissue-specific manner most notably in gliomas, and various types of skin cancer suggesting a potential role in the pathogenesis of these solid malignancies. Thus*, IDH1* and *IDH2* could be useful as molecular therapy targets. Furthermore, patients bearing the *IDH1* mutation can be benefitted from the ivosidenib or recently developed IDH1 mutant-specific peptide vaccine (IDH1-vac) and may also serve as diagnostic markers in these cancers. The *IDH1* and *IDH2* gene mutations can be used in clinical practice as strong prognostic biomarkers in gliomas as they could predict better survival.

This work represents an advance in biomedical science because it shows *IDH1* and *IDH2* mutational spectrum, significant prevalence in large cancer series and benefit of testing them for prognosis and therapeutic management.

## Summary Table

### What Is Known About This Subject?

• Isocitrate dehydrogenase genes, *IDH1* and *IDH2* have been demonstrated to be altered in brain cancers
• The *IDH1* and *IDH2* mutations were shown to predict the outcome of the patients with various brain malignancies
• Mutant IDH1 bearing gliomas can be therapeutically benefited from recently developed IDH1 mutant-specific peptide vaccine

### What This Work Adds

• Analyses of *IDH1* and *IDH2* mutations in large malignant series shows a complete spectrum of these mutations in human cancers
• The *IDH1* and *IDH2* somatic mutations play a significant role not only in brain tumors but also in other malignancies

## Data Availability

The original contributions presented in the study are included in the article/supplementary material, further inquiries can be directed to the corresponding author.
